# Development of Detection Method Using Dried Blood Spot with Next-Generation Sequencing and LabDroid for Gene Doping Control

**DOI:** 10.3390/ijms26136129

**Published:** 2025-06-26

**Authors:** Kiyoshi Maehara, Atsushi Hirokawa, Hinata Watanabe, Norihiro Otani, Jiawei Wan, Takanaga Shirai, Tohru Takemasa, Koichi Watanabe, Takeki Nishi, Ken Sato, Suzuka Shimmura, Kieu D. M. Nguyen, Yoichiro Takahashi, Takehito Sugasawa

**Affiliations:** 1 Doctoral Program in Medical Sciences, Graduate School of Comprehensive Human Sciences, University of Tsukuba, 1-1-1 Tennodai, Tsukuba 305-8572, Japan; 2 College of Medicine, School of Medicine and Health Sciences, University of Tsukuba, 1-1-1 Tennodai, Tsukuba 305-8572, Japan; 3 Doctoral Program in Sports Medicine, Graduate School of Comprehensive Human Sciences, University of Tsukuba, 1-1-1 Tennodai, Tsukuba 305-8572, Japan; 4 Laboratory of Sports Medicine, Department of Clinical Medicine, Institute of Medicine, University of Tsukuba, 1-1-1 Tennodai, Tsukuba 305-8572, Japan; 5 Institute of Health and Sport Sciences, University of Tsukuba, 1-1-1 Tennodai, Tsukuba 305-8574, Japan; 6 Department of Human Sciences, Kanagawa University, 3-27-1 Rokkakubashi, Kanagawa-ku, Yokohama-shi 221-8686, Japan; 7 Department of Legal Medicine, Institute of Medicine, University of Tsukuba, 1-1-1 Tennodai, Tsukuba 305-8575, Japan; 8 Human Biology Program, University of Tsukuba, 1-1-1 Tennodai, Tsukuba 305-8577, Japan

**Keywords:** gene doping, dried blood spot, erythropoietin, NGS, LabDroid, Maholo

## Abstract

In recent years, as gene therapy technology has rapidly developed, there has been growing concern that it could be misused by athletes as a means of doping. However, current testing methods for gene doping have a range of limitations and require further improvement. Furthermore, significant progress has been made in the fields of blood storage, next-generation sequencing (NGS), and LabDroid (experimental robots). Against this background, this study was implemented to develop a test method for gene doping using dried blood spot (DBS), NGS, and the LabDroid ”Maholo”. As a first step, recombinant adeno-associated virus containing the human erythropoietin gene (h*EPO*) was injected into mice to establish a gene doping model. Subsequently, DBS was created using whole blood. Maholo was used to extract DNA from the DBS and to create DNA libraries for NGS. NGS in combination with bioinformatic analysis clearly identified DNA fragments that provided definitive evidence of gene doping in the mouse model, which were absent in the control mouse. To the best of our knowledge, this is the first attempt to use a biological model of h*EPO* gene doping in conjunction with Maholo, NGS, and DBS. This method should facilitate the further development of gene doping tests.

## 1. Introduction

Doping refers to the use of substances or methods prohibited by the World Anti-Doping Agency (WADA) to enhance athletic performance [1]. Since its establishment in 1999, WADA has formulated anti-doping rules and policies for sporting competitions around the world. Through research and education, it rigorously pursues the goal of ensuring that all athletes can participate in fair competitions free from doping [2]. However, despite these efforts, doping remains a persistent issue. For example, in the lead-up to the Olympic Games Tokyo 2020, an American man was arrested on suspicion of distributing banned drugs to athletes [3]. Moreover, during the Olympics, it was discovered that a Bahraini track and field athlete had doped via a blood transfusion, which was made public [4]. Moreover, in November 2024, after the Olympics had finished, a test on a Japanese racewalker raised a suspicion of blood doping, with this athlete being suspended as a result [5].

Doping has continued to evolve along with medical advances, and the methods have become increasingly sophisticated. In particular, gene therapy technology has been developing rapidly. This technology has led to significant breakthroughs in the treatment of disorders such as muscular dystrophy, spinal muscular atrophy, and hemophilia [6,7]. However, as this technology develops, it raises growing concerns about gene doping in sports. Therefore, WADA chose to explicitly include gene doping in its Prohibited List and expand monitoring efforts [8,9]. Our research group has been investigating methods of detecting this form of doping for over 7 years [10,11,12,13,14,15].

One of the most concerning examples of gene doping is the introduction of the erythropoietin (EPO) gene. EPO as a recombinant protein has long been misused by athletes in various sports with the aim of improving endurance [16,17,18,19,20]. Meanwhile, in the field of gene therapy, it has recently been reported that recombinant adeno-associated virus (AAV) vectors are extremely safe, with many actual clinical trials having been performed [21,22]. This background suggests that there is a high risk of the combination of AAV vectors and *EPO* being misused for doping. This in turn highlights the urgency of developing a method for testing h*EPO* gene doping using this vector.

At the 2024 Paris Olympics, the International Testing Agency (ITA) introduced dried blood spot (DBS) sampling for doping tests and reportedly conducted gene doping analyses [23]. However, since the specific methods used have not been disclosed, it is possible that they are still in the development stage. Compared with conventional blood sampling, DBS is a minimally invasive technique requiring only a small amount of blood. Additionally, the stability of DBS at room temperature eliminates the need for frozen storage, significantly reducing logistical costs. For these reasons, it would be reasonable to use DBS in future gene doping tests.

As detection technologies advance, minimizing the risk of contamination remains a crucial challenge. Regardless of the sophistication of testing methodologies, the mishandling of samples can introduce contaminants, potentially leading to athletes being incorrectly accused of doping. One approach to mitigate this risk is the automation of sample preprocessing and DNA extraction using the humanoid laboratory robot (LabDroid) “Maholo” [24]. Our research group has employed Maholo for DNA or RNA extraction, and library preparation for next-generation sequencing (NGS). This robot is designed to handle samples of nucleic acids and, because it can perform experiments in an automated manner without human involvement, it can make a major contribution to preventing contamination and misconduct in the laboratory. In addition, this robot can be customized to suit researchers’ needs, making it applicable not only to nucleic acids but also to other applications. In practice, the robot has been able to automatically create pluripotent stem cells (iPSCs) [25] and iPSC-derived retinal pigment epithelial (iPSC-RPE) cells [26,27,28]. Furthermore, our research group has successfully utilized this robot in veterinary medicine research to create DNA libraries for NGS [28]. These factors suggest that Maholo would be particularly advantageous for gene doping tests. In addition, our research group has developed technology to comprehensively analyze DNA via bioinformatic analysis [28,29], which could also potentially be applied to gene doping tests.

Against the above background, the aim of this study is to develop a next-generation testing method by integrating DBS, Maholo, and NGS (as whole-genome sequencing, WGS), using a mouse model that expresses h*EPO* introduced by an AAV vector (AAV9_h*EPO*).

## 2. Results

### 2.1. Creation of a Gene Doping Model Using AAV9_hEPO

**Figure 1:**
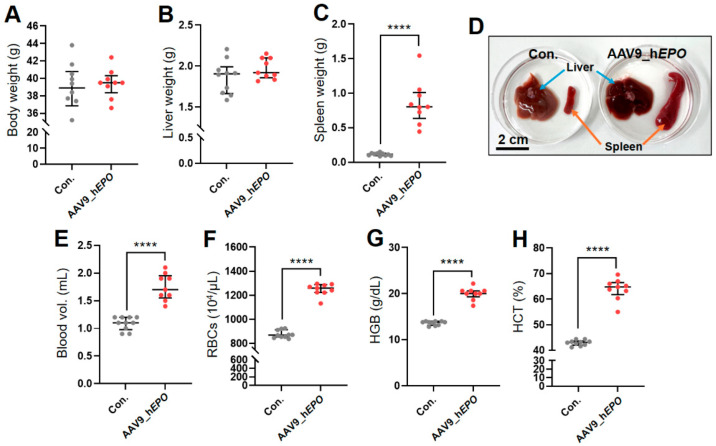
Establishment of mouse model of gene doping using AAV9_h*EPO*. (**A**) Body weight, (**B**) liver weight, (**C**) spleen weight, (**D**) representative image of liver and spleen samples, (**E**) blood volume, (**F**) RBCs, (**G**) HGB, (**H**) HCT. **** *p* < 0.0001.

AAV9_h*EPO* was injected into retro-orbital sinus in mice and, 10 days later, whole blood was collected and the mice were dissected. The results reveal no significant differences in body weight or liver weight between these mice and the control group (Con.) (Figure 1A,B). Meanwhile, in the AAV9_h*EPO* group, a significant increase in spleen weight was observed compared with Con (Figure 1C). The enlargement of the spleen was also confirmed macroscopically from a photograph of typical samples (Figure 1D). Furthermore, in blood tests, the levels of blood volume, red blood cells (RBCs), hemoglobin (HGB), and hematocrit (HCT) were significantly increased in the AAV9_h*EPO* group (Figure 1E–H). These results indicate that AAV9_h*EPO* led to the successful introduction, and that model mice of a gene doping with an increased blood volume were established as intended.

### 2.2. Gene Doping Was Detected from DBS Using TaqMan qPCR Assay

**Figure 2:**
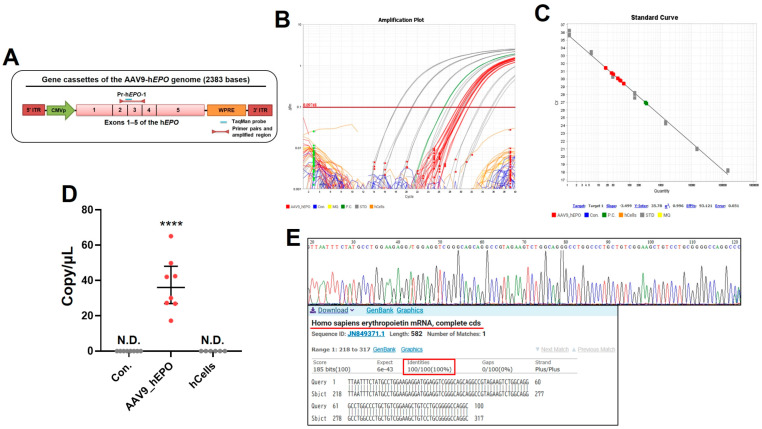
Proof of the gene doping detected by the TaqMan qPCR assay. (**A**) Primer/probe used in this experiment, (**B**) amplification curve (gray: standard sample; green: positive control; orange: cell lines as negative control; blue: Con. group; red: AAV9_h*EPO* group), (**C**) standard curve, (**D**) absolute quantification of DNA fragments as proof of the gene doping, (**E**) the waveform and sequence obtained by the Sanger sequencing of a representative sample, with alignment analysis. **** *p* < 0.0001 vs. other groups, N.D.: not detected.

After the animal experiment, the whole blood of the model mice was stored as DBS. To create negative control samples, suspensions of human cell lines were also dropped onto filter paper and stored. Samples with plasmids soaked into filter paper were also prepared as positive controls. Subsequently, DNA extraction from the DBS or filter paper and the preparation of reaction plates for the TaqMan qPCR assay were carried out using the automated Maholo program without human involvement. The design of the primer/probe to detect gene doping is shown in Figure 2A. The extracted DNA was judged to be suitable for the qPCR assays, despite some of the samples being of low quality (Supplementary Table S1). The qPCR assay results show that only the AAV_h*EPO* group and positive control showed positive amplification, while no amplification occurred in the Con. group or the human cell line as negative controls (Figure 2B). The standard curve for absolute quantification had an R^2^ of 0.996, which indicated accuracy (Figure 2C). When the absolute number of DNA fragments as evidence of gene doping was quantified using this calibration curve, it was found to be 17–65 copies/μL in the doped mice (Figure 2D). Sanger sequencing was performed for the amplicons of all samples in the AAV_h*EPO* group. The results confirm that the sequences were identical to the h*EPO* gene for all samples (Figure 2E). These results indicate the accurate detection of gene doping using qPCR. They also suggest that the automated program in Maholo worked without contaminating the samples.

### 2.3. Whole-Genome Sequencing and Bioinformatic Analysis Can Detect Gene Doping

**Figure 3:**
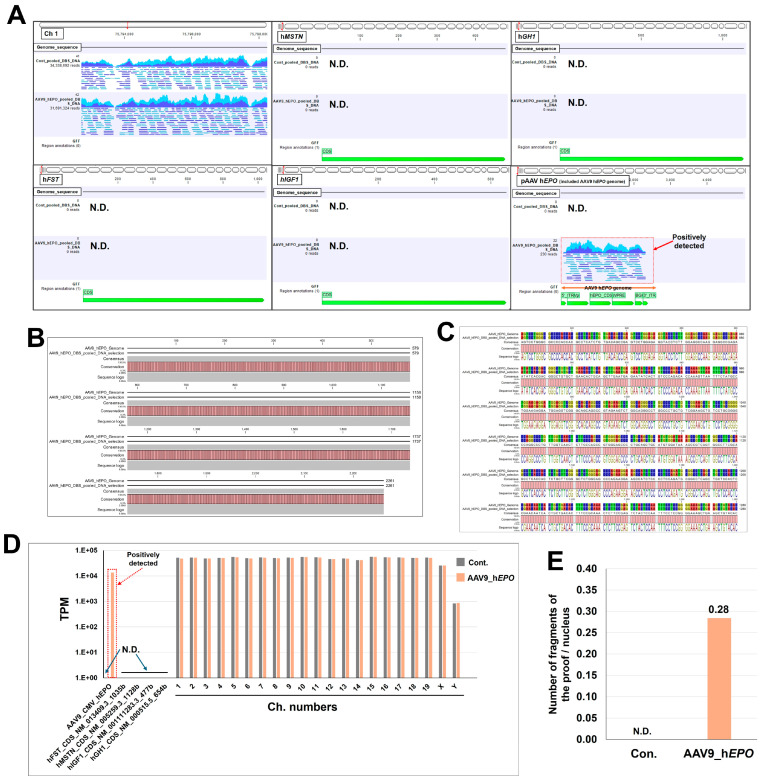
WGS and bioinformatic analysis make it possible to detect gene doping. (**A**) Visualization of reads mapped to each reference sequence (visualization of SAM files), (**B**) bar plot of alignment analysis of the sequence constituting evidence of gene doping and the reference sequence, (**C**) enlarged image of part of the alignment shown in **B** (bases 801 to 1280), (**D**) calculation of TPM values for each chromosome and gene set as references, (**E**) number of fragments of the proof per nucleus. N.D.: not detected.

The DNA extracted by the automated program was pooled by group. This pooled DNA was used as a template to create a library using the automated program. The library had an average size of 503–521 bp, which was suitable for WGS (Supplementary Figure S1). After sequencing, FASTQ files were obtained for bioinformatic analysis. In the bioinformatic analysis, negative and positive controls were set using a reference sequence that could be misused by athletes. Then, the detection of gene doping was performed. In the results, the mapped reads were confirmed in the chromosome region in both mouse groups, which is the positive control (Figure 3A). Meanwhile, the four gene regions set as negative controls (h*MSTN*: human myostatin; h*GH1*: human growth hormone 1; h*FST*: human follistatin; and h*IGF1*: human insulin-like growth factor) did not map in both mouse groups (Figure 3A). After establishing these negative and positive controls, many reads in the AAV9_h*EPO* group were mapped to the AAV vector sequence, providing proof of gene doping (Figure 3A). After extracting the sequence of the proof and converting it to the FASTA format, an alignment analysis was conducted with the AAV vector sequence, which matched at 100% (Figure 3B,C; Supplementary Figure S2). As the analysis up to this point was qualitative, a quantitative analysis was then conducted by calculating the transcripts per million (TPM) value, which is commonly used in RNA sequence analysis. The results similarly reveal the proof in the AAV9_h*EPO* group (TPM value of 13,654.2), with the results also confirming that the negative control and positive control were successfully established (Figure 3D). Furthermore, the number of DNA fragments per cell was calculated from the TPM value as proof of the gene doping. The results are N.D. for the Con. group and 0.28 for the AAV9_h*EPO* group (Figure 3E). These results suggest that WGS and a subsequent bioinformatic analysis are useful for detecting gene doping.

## 3. Discussion

Anti-doping tests using DBS have been considered advantageous in terms of collection, transport, and storage compared with the conventional use of whole blood [30,31,32]. Indeed, on 1 September 2021, WADA approved the use of DBS as a new sample matrix for doping tests [32,33]. Since then, studies have been conducted to confirm the usefulness of DBS for the testing of various prohibited compounds. Indeed, previous reports showed the usefulness of DBS in the testing of peptide hormones, stimulants, antianginal drugs, narcotic analgesics, and anabolic agents, among others [34,35]. Furthermore, a report has described that the sequence of h*EPO*, a simulated transgene, was efficiently detected from DBS using a TaqMan qPCR assay as a spike-in experiment using a plasmid [36]. However, because DBS testing requires only a small amount of blood, it has been asserted that efforts should be made to improve its detection sensitivity [35]. In any case, DBS is clearly useful for doping tests, and we hypothesized that it can also be applied to detect gene doping. We also considered that an analytical approach using robotics and NGS, which have been rapidly progressing in recent years, is highly compatible with gene doping tests; so, we combined these methodologies in this experiment. To the best of our knowledge, our results are the first example of the successful detection of gene doping in a biological model using a combination of DBS, the experimental robot Maholo (automated program), and NGS-based WGS. In the actual WGS data, positive and negative controls were also established, with the findings additionally proving the absence of contamination. These results suggest that doping can be detected even if the sample type is switched from whole blood to DBS, and also demonstrate the usefulness of WGS and Maholo. In addition, the model mice were administered a single dose of the AAV vector. Under conditions in which a sufficient dose was administered, both TaqMan qPCR and WGS enabled the precise detection of gene doping from DBS specimens in this study. Moving forward, it will be necessary to assess the detection sensitivity under more practical conditions, particularly with micro-dosing protocols. Under conditions in which detection using TaqMan qPCR or WGS becomes challenging, the integration of complementary approaches such as digital PCR or pre-concentration of DBS samples may prove beneficial.

Given the biological characteristics of the AAV vector used in this study, it is important to consider its persistence and detectability over time. AAV primarily exists in target organ cells in an episomal form [37], and its genomic integration frequency is extremely low, reportedly as little as 0.01% [38]. As a result, even if vector-specific DNA sequences are no longer detectable in blood samples, traces of gene doping in the form of episomal or integrated DNA may persist in target tissues. However, the direct analysis of such tissues is generally infeasible in humans. In our previous study using the same mouse model, we detected vector-derived fragments in whole blood up to 30 days post-administration [15], although their abundance declined over time. Therefore, detection becomes increasingly difficult beyond this window. These findings highlight the importance of estimating the likely timeframe of gene doping and performing multiple tests during the period when residual vector fragments are still detectable in the bloodstream.

The TaqMan qPCR assay used in this study is inexpensive, but a single-plex assay can only detect one DNA sequence. By contrast, WGS using NGS can detect everything at once, which may be important for future gene doping tests. One of the issues with WGS in gene doping tests is that it is inefficient in terms of data analysis costs and computational load, as the majority of sequence reads are derived from nuclear DNA. However, WGS has the main advantage that it enables comprehensive analysis. Moreover, the read data as a FASTQ file obtained by NGS can be stored semi-permanently under appropriate management like that at European sequencing laboratories [39], enabling their re-examination on a computer even 10 years later. This means that, even if athletes dope with unknown genes to enhance their performance, it will be possible to re-examine them at a later date by adding a reference sequence. Regardless of these benefits and disadvantages, the use of NGS in gene doping testing will probably advance dramatically if future research develops an assay that can selectively remove unnecessary chromosomal DNA before sequencing.

There are still some technical issues to be addressed in this study. The three processes of DNA extraction and purification, plate preparation, and NGS library preparation were carried out by the automated program of Maholo, effectively reducing the risk of contamination [24]. However, punching out the DBS and preparing homogenate solutions, as well as measuring DNA concentrations, still needed to be performed manually. As these steps are potentially associated with the risk of contamination, further improvements are needed to achieve full automation. Furthermore, this research involved an experimental system using model mice. Further verification is thus needed before this approach can be applied to the testing of actual athletes. More specifically, there is a need to verify in detail whether the series of methods used in this research can be applied to samples from patients who have received gene therapy. In addition, when acquiring human gene doping-related data, sufficient care is needed to prevent the leaking of sensitive information such as medical conditions and genetic background.

In recent years, concerns about gene doping in racehorses have increased, as they have in humans, for which the development of testing methods has progressed rapidly. As with humans, there are concerns about the misuse of genes that improve athletic performance, including *EPO*, *MSTN*, *FST*, *GH1*, *VEGF*, and *IGF1* [40,41]. Furthermore, unlike in humans, there is a growing risk of doping through the genetic manipulation of fertilized eggs or embryos in horses, and there is an urgent need to develop testing methods for this [42]. A Japanese group conducting pioneering research into gene doping tests of racehorses has developed a variety of methods with aim of eradicating doping, which they are continuously innovating. This group has demonstrated that it is possible to detect transgenes with high sensitivity using πCode technology, droplet digital PCR (ddPCR), and microfluidic quantitative PCR [43,44,45,46]. They further concluded that transgene detection by WGS would be possible even when degraded DNA from horse hair root was used as a template [47]. In addition, in a unique experiment, they also developed a method for detecting transgenes using direct ddPCR and nested ddPCR, which does not require DNA extraction and purification; this approach is expected to be applied to rapid testing in the field [48]. The group has also shown that it is possible to detect transgenes as pseudogenes using the FASTA file of a horse obtained from WGS and bioinformatic analysis software called DELLY [49], suggesting the possibility of applying this method to the examination of gene doping through fertilization or high-level manipulation [50,51,52]. At the same time, because pseudogenes exist at a certain rate, PCR testing alone may also produce potential false positives, highlighting the importance of using both PCR testing and WGS [51]. The various methods that they have developed may be applied flexibly in a variety of ways according to the situation in the field of genetic doping tests for racehorses. Therefore, we should also refer to their methods in human testing. In this study, we used their methods and findings as a reference, and performed qPCR assay, WGS, and subsequent bioinformatic analysis to detect gene doping. The results reveal that doping was detected using both methods, limiting the possibility of false positives and ensuring accuracy and robustness. However, in this experiment, we did not use DELLY, which they recommend. In future research, it would be desirable to use DELLY to exclude false positives in addition to the bioinformatic analysis used in this study.

## 4. Materials and Methods

### 4.1. Graphical Presentation of Experimental Methods

**Figure 4:**
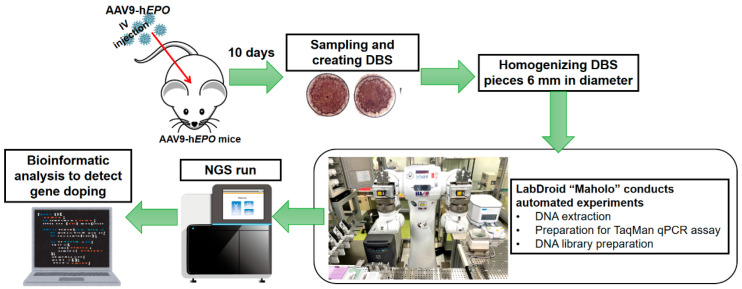
An overview of the experimental procedure.

An overview of the experimental procedure is shown in Figure 4. The procedure is shown in a series of steps: creating gene doping model mice, creating DBS, extracting and purifying DNA using Maholo, TaqMan qPCR assay, NGS run, and bioinformatic analysis.

### 4.2. Creation of the AAV9_hEPO Vector

The AAV9_h*EPO* vector was created using the method we previously reported [15,53]. In brief, this vector was designed as a plasmid by connecting h*EPO*, the CMV promoter, and other functional sequences using a platform [54] operated by Vector Builder Inc. (Science City, Guangzhou, China). After design, we asked the company to amplify and purify AAV9_h*EPO*. The concentration of AAV9_h*EPO* delivered to our laboratory was 3.29 × 10^13^ genome copies (GC)/mL.

### 4.3. Animal Experiments

The animal experiments conducted in this study were approved by the Animal Care Committee of the University of Tsukuba (approval number 24-106). Nineteen mice were purchased from CLEA Japan (Tokyo, Japan) at the age of 6 weeks and then underwent a 1-week acclimation period. The mice were healthy and had not received any prior treatment. The mice were bred and maintained under specific pathogen-free conditions in an air-conditioned animal house. The animals were subjected to a 12/12 h light/dark cycle with standard mouse pellets and water provided ad libitum. Upon the commencement of the experiments, the mice were 7 weeks old and weighed 36.7 ± 2.0 g (mean ± SD). After the 1 week of acclimation, the mice were randomly assigned to the control group (*n* = 10; designated as “Con.”) or the AAV9_h*EPO* group (*n* = 9; designated as “AAV_h*EPO*”) (simple randomisation). Mice in the same group were housed together. In other words, the Con. and AAV_h*EPO* groups were kept separate. This was conducted to prevent the Con. mice from being contaminated by the virus vector, which could lead to inaccurate analysis.

All subsequent treatments and analyses were performed in the following order: Con., followed by AAV_h*EPO*. The procedures were conducted in order of mouse number, starting with the lowest number. Additionally, the experimenters were informed of each mouse’s number and group assignment. Therefore, potential confounding factors were not considered in any analysis.

The mice in the AAV_h*EPO* group received a retro-orbital sinus injection of the AAV9_h*EPO* vector at a dose of 10^11^ GC/100 µL/mouse under systemic isoflurane anesthesia. The mice in the control group received injections of the 10% glycerol/PBS buffer (100 µL/mouse) used to suspend the AAV9_h*EPO* vector. This dosage was determined based on our previous research, which may be a reasonable dosage even for humans [15]. Ten days later, under general anesthesia with isoflurane inhalation, whole blood was collected from the inferior vena cava using EDTA-2Na as an anticoagulant. The amount of blood that could be collected was recorded at that time. Subsequently, under general anesthesia, the mice were euthanized by cervical dislocation. After euthanasia, the liver and spleen were collected, weighed, and photographed [15].

### 4.4. Blood Test

Using 50 μL of the obtained whole blood, the number of RBCs, HGB level, and HCT value were measured using an automated blood analyzer (Celltac α MEK6458; NIHON KODEN, Tokyo, Japan).

### 4.5. Preparation of DBS and Homogenate

A total of 150 μL of whole blood was dropped onto filter paper and dried at room temperature for 1 h in a clean bench to prepare DBS. It was then stored with silica gel at −20 °C for 3 months. To prepare samples that would serve as negative controls, the suspensions as 2 × 10^6^ cells/mL PBS of six human cell lines (hCells) (fibroblasts, keratinocytes, liver cancer cells, stomach cancer cells, ovarian cancer cells, and kidney cells) were also dropped in the same way, and dried cells on filter paper were also prepared. On the day of the experiment to extract DNA, three circular pieces were punched out of the DBS using a 6-millimeter-diameter biopsy punch for each sample. These pieces were placed in a 2 mL microtube, and then 500 μL of lysis buffer (Cat#NPK-101, MagExtractor Genome; TOYOBO, Tokyo, Japan) was added along with two pieces of crushing beads to physically crush the sample to make a homogenate. Next, the supernatant of the homogenate was obtained by centrifugation at 12,000× *g* for 10 min at room temperature. The supernatant was then centrifuged again under the same conditions to obtain a supernatant free of impurities. A total of 170 μL of the supernatant was applied into a well of a 96-well plate, after which the plate was set on the experimental rack of Maholo.

### 4.6. DNA Extraction and Preparation of TaqMan qPCR Assay Using Maholo

DNA extraction and the preparation of the TaqMan qPCR assay reaction plate were carried out using an automated Maholo program. Owing to the limited number of wells in the reaction plate, the number of samples in each group was set to 8. When selecting these samples, eight were selected in order of the smallest numbers assigned to mice from each group. The program was created in advance and confirmed to be accurate (Supplementary Figure S3A). The reagent kit used in the DNA extraction and purification process of this program was MagExtractor Genome (TOYOBO). Finally, the DNA from the DBS was dissolved in 30 μL of pure water. The TaqMan qPCR assay reaction plate was prepared by Maholo as follows: PrimeTime Gene Expression Master Mix (Cat# 1055771; Integrated DNA Technologies, Coralville, IA, USA) and a primer/probe specific for h*EPO* (overlapping exons 2–4; synthesized by Integrated DNA Technologies) that does not take introns into account were used to create the reaction plate. The design of the primer/probe was based on WADA’s laboratory guidelines [55] and is intended to detect h*EPO* gene doping. In the preparation, the primer concentration was 500 nM and the probe concentration was 250 nM, and the amount of template DNA (undiluted solution) was 3 μL, with a final volume of 15 μL/well. The primer and probe sequences are shown in Supplementary Table S1. Wells for the creation of a standard curve were also established to enable absolute quantification, using pAAV_CMV_h*EPO* (the plasmid for constructing the AAV vectors). The range of the standard curve was set from 147,324.01 to 1.17 copies/μL. After preparing the reaction plate, the 96-well plate containing the DNA extracted by Maholo was removed from the experimental rack in Maholo, and the DNA concentration was measured manually using NanoDrop One (Thermo Fisher Scientific, Waltham, MA, USA) (Supplementary Table S2).

### 4.7. TaqMan qPCR Assay to Detect Gene Doping

TaqMan qPCR assay was conducted on QuantStudio 1 Real-Time PCR System (Thermo Fisher Scientific) as absolute quantification. This qPCR assay was carried out on equipment outside Maholo’s laboratory space. The thermal cycling conditions consisted of initial denaturation at 95 °C for 5 min, followed by 40 cycles of denaturation at 95 °C for 2 s and annealing/extension at 60 °C for 20 s. The standard curves generated from these assays demonstrated a coefficient of determination (R^2^) exceeding 0.99, affirming the assay’s precision [15].

### 4.8. Sanger Sequencing

The amplicons obtained from the TaqMan qPCR assay were purified using the NucleoSpin Gel and PCR Clean-up kit (Cat# 740609; Takara Bio, Kusatsu, Japan). Subsequently, using 2 ng of the purified amplicon DNA, Sanger sequencing was outsourced to GENEWIZ (Tokyo, Japan). The primers used are shown in Supplementary Table S1. The waveform and sequence data are attached in Supplementary Data S1. The data were analyzed using Nucleotide BLAST [56], and an alignment analysis was performed.

### 4.9. WGS

WGS was performed to detect gene doping. First, the DNA extracted from DBS was pooled for each group of eight mice and adjusted to 5 ng/μL. The library was prepared using the NEBNext Ultra II FS DNA Library Prep Kit for Illumina (Cat#E7805S; New England Biolabs, Ipswich, MA, USA) and the automated program on Maholo, using 50 ng of pooled DNA. The fragmentation time was set to 5 min, and the final number of PCR cycles was set to 6. The automated program on Maholo was created in advance and confirmed to be free of errors (Supplementary Figure S3B). After the run on Maholo, the concentration and size distribution of the library were confirmed using an Agilent 2100 Bioanalyzer system (Agilent Technologies, Santa Clara, CA, USA) with Agilent DNA 7500 Kit (Cat#5067-1506; Agilent Technologies), outside of Maholo’s laboratory space. Libraries with an average size of 503–521 bp were created in each group (Supplementary Figure S1). These libraries were pooled and adjusted to 10 nM. The adjusted libraries were then sent to Novogen (Tokyo, Japan), where they were sequenced as paired-end 150-bp reads using the company’s NovaSeq X Plus (Illumina, San Diego, CA, USA) and a 10B flow cell (Illumina). Finally, the BCL file was obtained and FASTQ data were generated using bcl2fastq2 Conversion Software v2.19.0 (Illumina) from the BCL file.

### 4.10. Bioinformatic Analysis

A bioinformatic analysis to detect gene doping was performed using CLC Genomics Workbench (ver. 24.0; QIAGEN, Hilden, Germany). The CLC software tool “QC for Sequencing Reads” was run to check the quality of the sequences. The results confirmed that 1.35 billion reads were obtained for the Con. sample and 1.17 billion reads were obtained for the AAV_h*EPO* sample. Next, the CLC software tool “Trim Reads” was run with the default settings to remove reads with low scores. After that, “QC of sequence reads” was run again, and approximately 0.02% of the reads were deleted. The number of reads used in the analysis was 13.50 billion for the Con. sample and 11.78 billion for the AAV_h*EPO* sample (in terms of bases, the numbers were 196.5 Gb and 171.5 Gb, respectively). The FASTA file used as a reference for the detection of gene doping was created using EmEditor (Emurasoft, Inc., Tsukuba, Japan). The FASTA file for GRCm39 [57] was used as a reference, and the sequence for pAAV_CMV_h*EPO* was added to it. In addition, the cDNA sequences of h*MSTN* (human myostatin) [58], h*GH1* (growth hormone 1) [59], h*FST* (follistatin) [60], and h*IGF1* (insulin-like growth factor 1) [61] were also added as negative controls, which are genes for which there is concern about potential misuse. The CLC software tool “Map Reads to Reference” was run using the trimmed reads with reference to the original FASTA file. With regard to the execution parameter settings, these were set so that only the reads that matched the reference sequence 100% were retained. The mapped reads as a SAM file with each reference sequence were visualized using the CLC software by executing “Create Track List”. For absolute quantification, TPM values were calculated using Microsoft Excel for Microsoft 365 ver. 2502 (Microsoft, Redmond, WA, USA) based on the counts mapped to each reference sequence. The sequence acting as evidence of gene doping was extracted by running the “Extract Consensus Sequence” tool in the CLC software. The sequence was then run through the “Create Alignment” tool in the CLC software to check whether it matched the sequence of pAAV_CMV_h*EPO*.

### 4.11. Statistical Analysis

Statistical analyses were performed on the data for the blood parameters and qPCR assays. GraphPad Prism version 10.2.0 (GraphPad, San Diego, CA, USA) was used for this analysis. First, the experimental data were evaluated using the Shapiro–Wilk normality test to confirm the normality of the distribution. Given that the data were found not to be normally distributed, a non-parametric test was selected for testing all of the data. Specifically, Wilcoxon signed-rank sum test was performed. A *p*-value less than 0.05 was considered to indicate statistical significance. The graphs with plots are depicted as individual values with medians and interquartile ranges.

## 5. Conclusions

This study presents a novel, integrative approach to detecting gene doping that combines DBS sampling, LabDroid Maholo automation, and NGS with bioinformatic analysis. This method successfully detected AAV-mediated h*EPO* transgenes in a mouse model, highlighting the potential of DBS-based genomic analysis in anti-doping science and contributing to the advance of minimally invasive, contamination-resistant, and automation-compatible doping control technologies. Future refinements, including improved sensitivity and validation in human subjects, are expected to enhance the method’s applicability.

## Data Availability

The original contributions presented in this study are included in the article and Supplementary Materials. Currently, our research team is performing secondary analysis of the raw NGS data (FASTQ, BAM, BCL files). There is a possibility of new discoveries in the future; so, we cannot release the data to ensure priority. However, if individual researchers clearly state the purpose of their use, we may distribute the data as needed. Further inquiries can be directed to the corresponding author.

## Supplementary Materials

The following supporting information can be downloaded at: https://www.mdpi.com/article/10.3390/ijms26136129/s1.
