# Supplementary material for: Development of Detection Method Using Dried Blood Spot with Next-Generation Sequencing and LabDroid for Gene Doping Control
Source: Int J Mol Sci. 2025 Jun 26;26(13):6129. doi: 10.3390/ijms26136129 (PMC12250089; doi:10.3390/ijms26136129)
Supplement: Supplementary file 1 [file ijms-26-06129-s001.zip › Figure S3 The automated programs on the Maholo.pptx]

## Slide 1
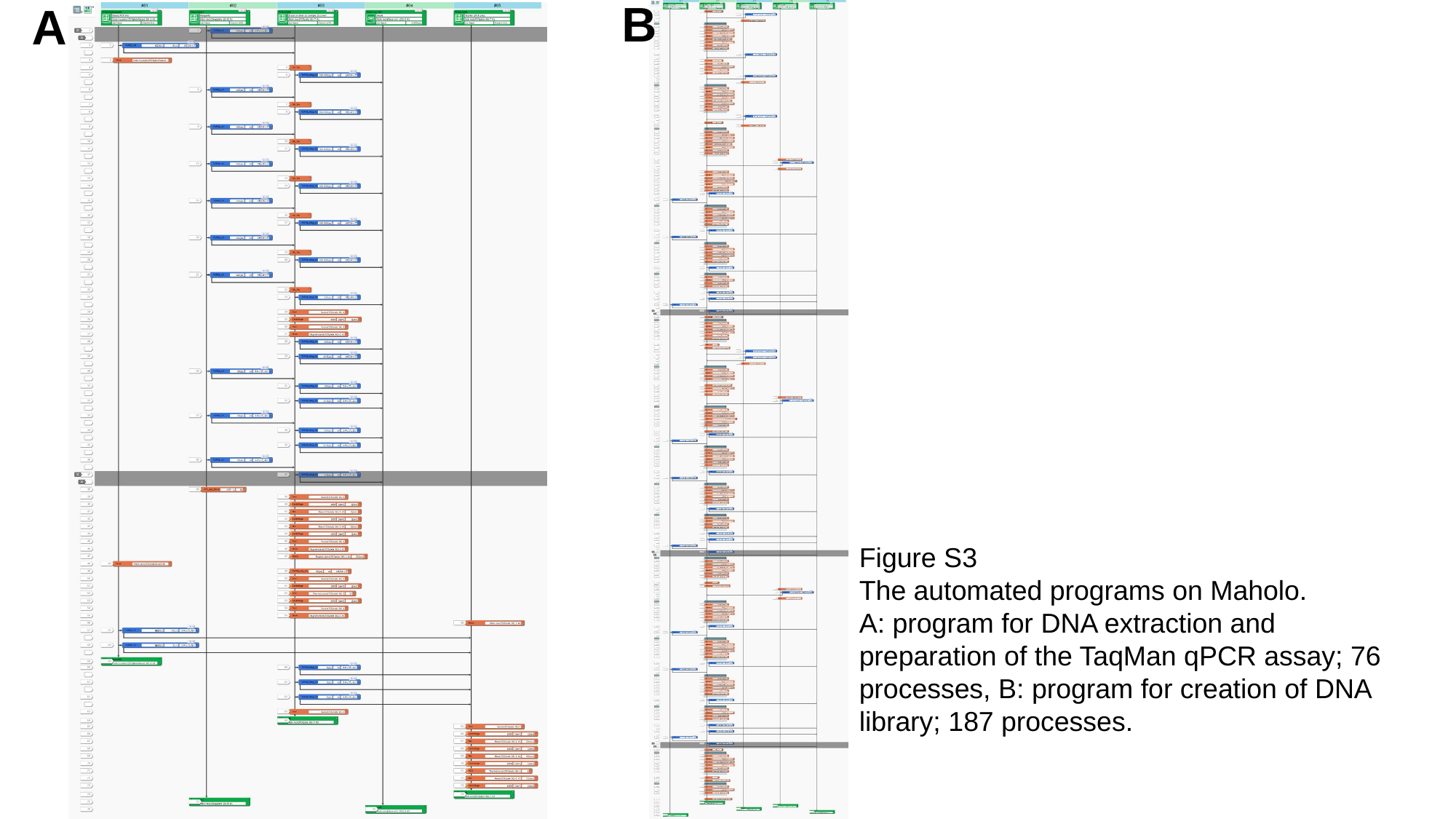

B
A
Figure S3
The automated programs on Maholo.
A: program for DNA extraction and preparation of the TaqMan qPCR assay; 76 processes, B: program for creation of DNA library; 187 processes.
