# Supplementary figures and images for: Development of Detection Method Using Dried Blood Spot with Next-Generation Sequencing and LabDroid for Gene Doping Control
Source: Int J Mol Sci. 2025 Jun 26;26(13):6129. doi: 10.3390/ijms26136129 (PMC12250089; doi:10.3390/ijms26136129)

## Slide 1
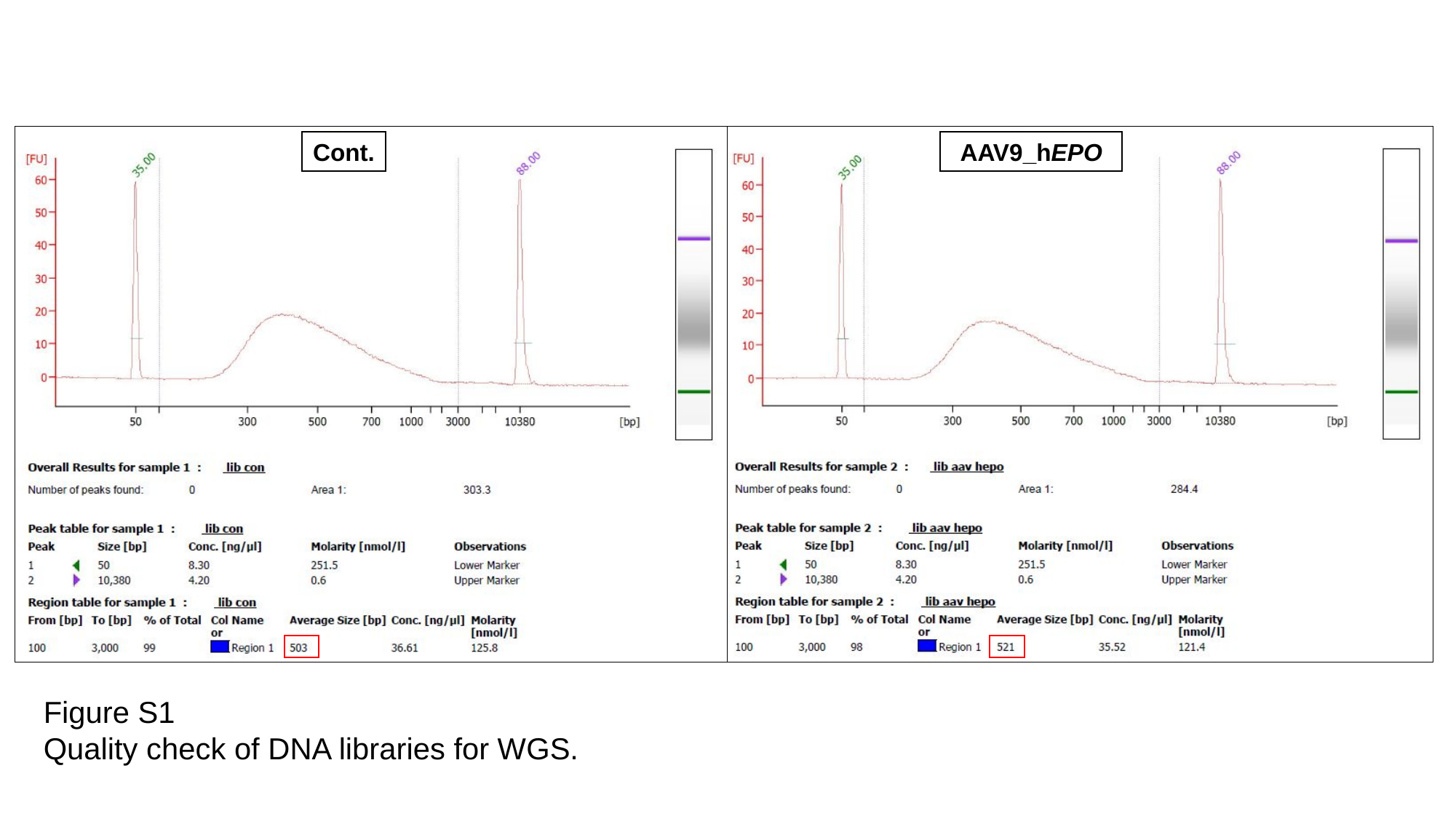

Cont.
AAV9_hEPO
Figure S1
Quality check of DNA libraries for WGS.

Supplement: Supplementary file 1 [file ijms-26-06129-s001.zip › Figure S1 Quality check of DNA libraries for WGS.pptx]

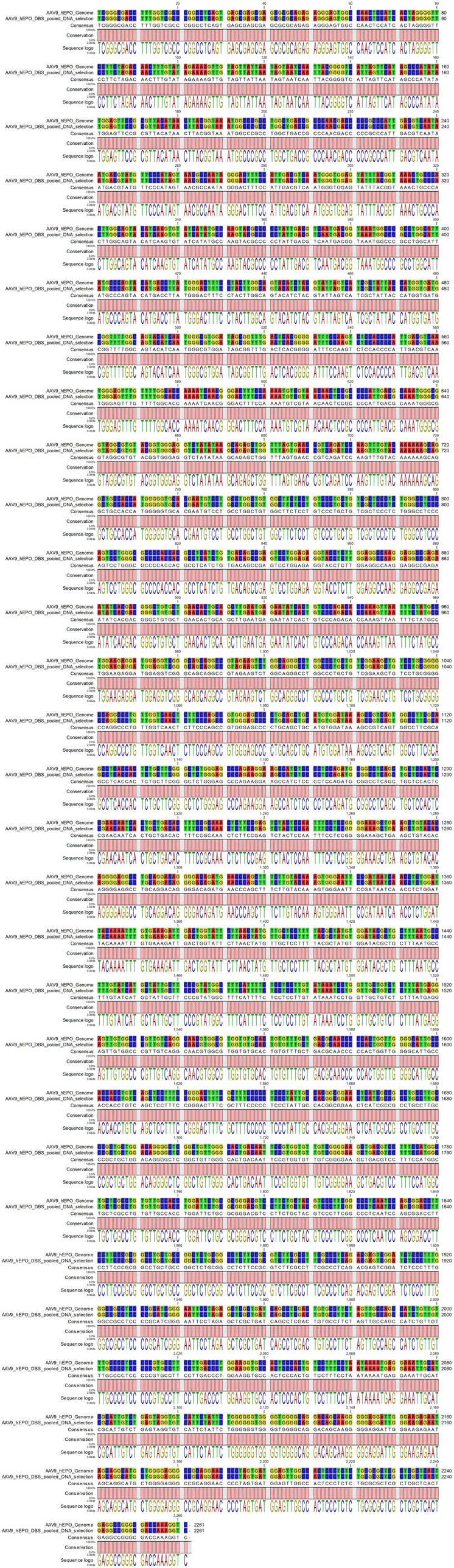

Supplement: Supplementary file 1 [file ijms-26-06129-s001.zip › Figure S2 Details of the alignment analysis.jpg]
